# Access to Hematopoietic Stem Cell Transplant in Canada for Patients with Acute Myeloid Leukemia

**DOI:** 10.3390/curroncol29080412

**Published:** 2022-07-22

**Authors:** Oluwatobiloba Morakinyo, Oliver Bucher, Kristjan Paulson

**Affiliations:** 1 Max Rady College of Medicine, University of Manitoba, Winnipeg, MB R3E 0W2, Canada; 2 Department of Epidemiology and Cancer Registry, CancerCare Manitoba, Winnipeg, MB R3E0V9, Canada; obucher@cancercare.mb.ca

**Keywords:** hematopoietic stem cell transplant, health services, allogeneic stem cell transplant, acute myeloid leukemia

## Abstract

Hematopoietic stem cell transplant is a complicated intervention only offered in specialized centers. Access to transplants may vary based on the location of primary residence, income levels, age, and reported race or ethnicity. Using data from the Canadian Institute of Health (CIH) Discharge abstract database (DAD), all non-Quebec Canadians under the age of 65 with a diagnosis of AML between 2004 and 2015 were included in this study. Descriptive statistics were produced for the variables of interest: time period, age, sex, rurality, transplant status, proportion of visible minorities, proportion identifying as indigenous, and proportion of low-income families. Transplant rates were compared and reported using univariable and multivariable analysis. In multivariable analysis, time period, province of residence, gender, and age were significantly associated with the receipt of an allogeneic hematopoietic stem cell transplant. However, differences in transplant rates observed in indigenous patients, low-income families, and visible minorities were not found to be statistically significant. In non-Quebec Canada, transplant rates vary significantly with province of residence, with the highest rates recorded in Alberta. Contrary to findings previously reported in studies exploring access to transplant in the United States, a low-income level was not associated with lower rates of transplants. This might suggest that Canada’s universal health care insurance program is protective against socioeconomic barriers that impact access to health care services.

## 1. Introduction

Allogeneic hematopoietic stem cell transplant (alloSCT) is a potentially curative treatment for patients with blood cancers and disorders of the blood/immune system, but due to the complexity of the procedure, it is only offered in a limited number of medical centers. Many barriers that prevent patients from receiving an alloSCT might exist, including physical and social geographic barriers, such as a rural location of residence, geographical distance from a transplant specialized center, the level of income, and race/ethnicity amongst others. While the literature and data on access to stem cell transplants in Canada is limited, studies on access to solid organ transplants have been conducted in Canada. One study conducted by Mucsi et al. [1] showed that African or Asian ethnicity was associated with lower access to kidney transplant. Another Canadian study examining the geographical disparities in access to liver transplantation in Atlantic Canada found that interprovincial disparities in access exist [2]. Beyond the Canadian border, several studies on access to alloSCT in the United States have been conducted over the years, and disparities in access have been identified among certain groups.

A literature review on access to hematopoietic cell transplantation in the United States conducted by Majhail et al. [3] identified age, sex, race, insurance status, and other barriers as specific disparities to access. One study examining geographical access to hematopoietic cell transplantation services in the United States showed that although nearly 94% of residents were within a three-hour distance of an age-appropriate transplant facility, some variations did exist with respect to age, race, and state of residence [4]. Another study looking at access to alloSCT for patients with Acute Myeloid Leukemia (AML) in Virginia showed that the rate of alloSCT varied with geographic regions within the state; furthermore, areas with higher percentages of non-Hispanic black or African American population had lower rates of receiving an alloSCT [5]. Similar findings were also reported in a study conducted by Jabo et al. [6], showing that AML and Acute Lymphocytic Leukemia (ALL) patients with Hispanic or black race/ethnicity predicted lower rates of alloSCT.

A more recent study on access to alloSCT using data from the Surveillance, Epidemiology and End Results (SEER) Program and the Center for International Blood and Marrow Transplant Research (CIBMTR) showed that higher levels of poverty were associated with lower rates of transplants [7]. Pidala et al. [8] examined practice variation in physician referral for alloSCT and found that physical referral decisions were largely impacted by insurance coverage, which served as a fundamental barrier in accessing transplant services. Given the differences that exist in the funding of the healthcare systems in the United States compared to Canada, we sought to understand how access patterns to alloSCT vary in Canada, a country with a government-funded universal health care system.

Previous studies done in Canada examining access to stem cell transplantation found that rural patients were somewhat less likely to receive an autologous hematopoietic cell transplant when compared to patients in urban areas; however, the difference identified was not statistically significant due to the small population size studied [9]. Recent literature out of Canada examining factors impacting access to alloSCT in the pediatric populations found no association between race, neighborhood income quintile or region, and receipt of transplant [10]. Another study specifically looking at access to alloSCT in pediatric populations with AML in Canada also showed equitable access within the publicly funded health care system [11]. To our knowledge, no studies examining what barriers exist for adult patients accessing alloSCT at the national level have been conducted in Canada; therefore, this study is the first to identify the barriers that exist and how they impact access to alloSCT in Canada.

## 2. Methods

The Canadian Institute of Health Information (CIHI) Discharge Abstract Database (DAD) is a national record of all hospital admissions in all Canadian provinces other than Quebec. All acute care facilities or their respective regional health authorities in all provinces and territories except Quebec are required to report data, which capture administrative, clinical, and demographic information of hospital discharges, to the DAD. The rate of transplant was defined as the number of alloSCTs performed by geographic area divided by the population. In other words, the transplant rate is the percentage of patients diagnosed with AML who went on to subsequently receive an alloSCT during the study period.

All Canadians under the age of 65 admitted to hospital in provinces other than Quebec with a diagnosis of AML between 2004 and 2015 were included in this study. Of the patients identified, we determined which of them were subsequently admitted for an alloSCT. The reason for including only patients under 65 is that many older patients who are diagnosed with AML are treated with chemotherapy regimens that do not always require hospitalization.

The area of residence of the included patients at the time of admission with AML was identified using their forward sorting area (FSA) component of the postal code. The second character of the FSA was used to determine rurality; with “0” defined as rural, and other characters defined as urban. In addition, the socio-demographic attributes of their area of residence using data from the 2006 Canadian Census were considered. These attributes included whether they resided in a rural or urban setting and what percentage of the population belonged to a visible minority group or indigenous group. The proportion of individuals belonging to a low-income group after tax was also included as a variable of interest.

Logistic regression was used to investigate potential associations between the variables of interest and the odds of receiving a transplant. To account for any difference in outcomes between the pediatric and adult population, sensitivity analyses were conducted separately for three different cohorts: (1) Full cohort (all patients), (2) Pediatric cohort (under 19 years old), and (3) Adult cohort (19 years old and above). Individuals from New Brunswick were excluded from the analyses due to a significant proportion of patient referrals to Quebec.

Descriptive statistics were produced for the variables of interest (time period, province, sex, age, rurality, transplant status, proportion of visible minorities, Indigenous, and proportion of low-income families after tax). Time period refers to the year in which the transplant was performed and was included to account for shifts in clinical practice over time. Significant differences between these variables among individuals that did and did not receive transplant were determined using Chi-squared and Wilcoxon–Mann–Whitney tests where appropriate. A significance level of 0.05 was chosen as the cut-off for significance. SAS and STATAMP were used for statistical analysis.

The variables of interest were screened for inclusion in a multivariable logistic regression model by testing univariable associations among all variables and the primary outcome of interest: receipt of transplant or not. Likelihood ratio testing was used to test these associations, with *p*-values of ≤0.2 indicating significance. All variables with *p*-values of ≤0.2 were further screened for autocorrelation using Cramer’s V, Pearson or Spearman correlation coefficients, and, where appropriate, point-biserial correlation and eta-squared. Pearson or Spearman coefficients greater than 0.80, Cramer′s V above 0.35, point-biserial correlations with *p*-values ≤ 0.05, and eta-squared > 0.26 were interpreted as indicative of potential autocorrelation.

The multivariable model was constructed from the remaining variables using likelihood ratio testing with *p*-values ≤ 0.05 indicating significance. The relationship among continuous variables was modeled using restricted cubic splines. Akaike’s information criteria was used to select models with different numbers of knots. Model fit was evaluated using the Hosmer–Lemeshow goodness-of-fit test. Outlying covariate patterns and those with large leverage and influence (delta-beta) were evaluated visually with scatterplots. 

## 3. Results

There were 6119 non-Quebec Canadians admitted to the hospital with a diagnosis of AML between 2004 and 2015. Overall, 1754 (28.7%) of those admitted with AML proceeded to receive an alloSCT. In multivariable analysis, time period, province of residence, gender, and age remained significantly associated with receipt of alloSCT. Table 1 highlights the descriptive characteristics for the variables of interest, while Table 2, Table 3 and Table 4 show the univariable and multivariable logistic regression models for the three cohorts included in this study.

As the time period increased, the predicted probability of transplants increased up to 2013, after which the probability leveled off. Although living in a rural vs. an urban location of residence was not significantly associated with transplant rate, the province of residence had a significant association with the odds of receiving a transplant. In comparison to the province of Alberta, residents from other Canadian provinces had lower odds of receiving a transplant. The differences were statistically significant in all instances except for the three territories and in Prince Edward Island. 

**Figure 1:**
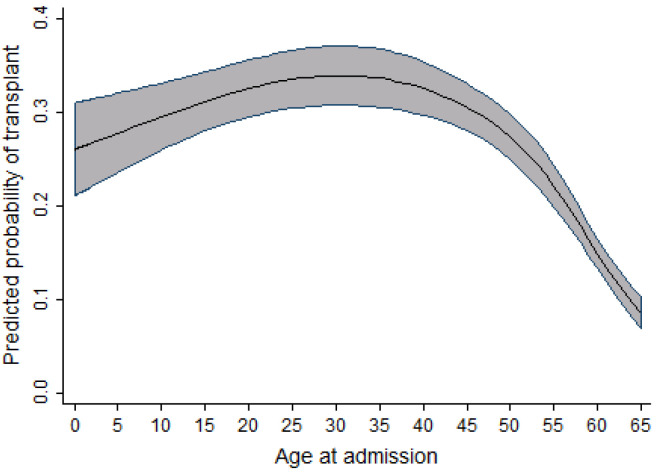
Relationship between age at admission and the probability of transplant (full cohort).

**Figure 2:**
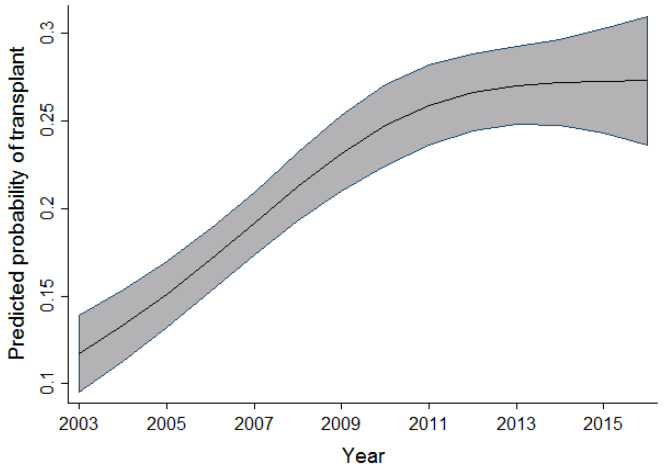
Relationship between time period and the probability of transplant (full cohort).

In univariable analysis, the effect of sex appeared to be highly significant; however, after taking into account the effects of time period, age, and province, this significance slightly reduced. When compared to men, the odds of receiving a transplant in women were observed to be 1.12 times higher. As the age of the patient increased, there was a slight increase in the probability of receiving a transplant. This effect was observed up to the age of 30, after which the probability of receiving a transplant decreased. In the adult cohort, as age increased, the probability of receiving a transplant decreased slightly between the ages of 19 and 50 years old. After the age of 50, a dramatic decrease in the probability of receiving a transplant was observed. Within the same cohort of adult patients, as the year of admission for transplant increased, the probability of receiving a transplant also increased. This effect was most prominent between 2003 and 2010. Figure 1 and Figure 2 display the relationship between the probability of transplant and the age and time period in the full cohort.

**Figure 3:**
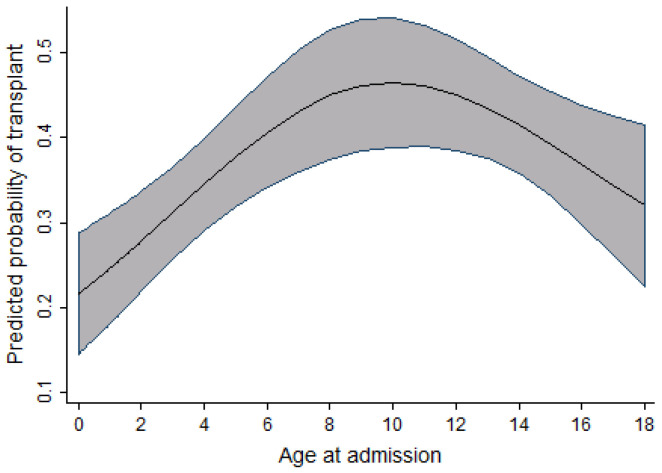
Relationship between age at admission and the probability of transplant (pediatric cohort).

**Figure 4:**
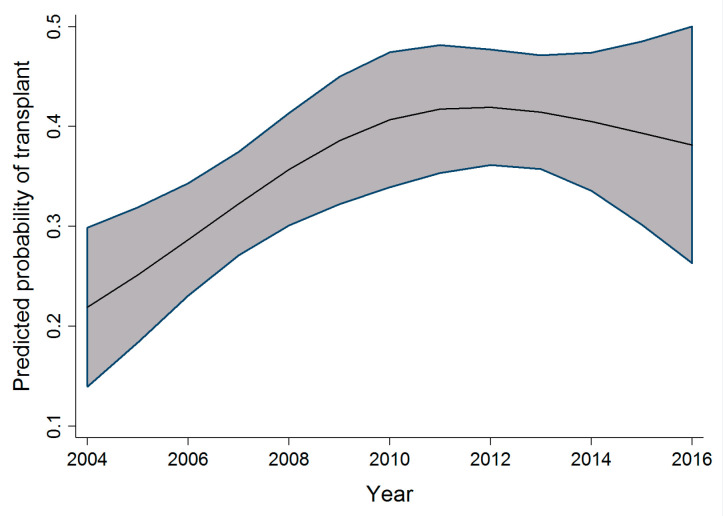
Relationship between time period and the probability of transplant (pediatric cohort).

In the pediatric cohort, as the age at admission increased, the probability of receiving a transplant also increased; however, this increase peaked at age 10, followed by a slow decrease in probability of receiving a transplant. Within the same cohort, an increase in the probability of receiving a transplant was observed to increase as the time period during which individuals were admitted for transplant increased. This relationship is seen from 2004 to 2010–2011, after which the probability reaches a plateau and then decreases. Figure 3 and Figure 4 display the relationship between the probability of transplant and the age and time period in the pediatric cohort.

The rate of transplants was not found to be significantly different in indigenous patients compared to non-indigenous patients in the full and adult cohorts. Similarly, there was no significant difference in transplant rates among low-income families. Overall, adult sensitivity analysis results were identical to the main cohort; however, in the pediatric subgroup, age and time period were the only variables with significant difference between transplant and non-transplant recipients.

## 4. Discussion

Allogeneic hematopoietic stem cell transplant is considered the preferred type of post-remission therapy in patients with AML; for many, it is the only curative therapy [12,13]. This study is the first in Canada to explore the sociodemographic variables that impact access to transplant, at the national level, in patients diagnosed with AML. Although the literature on sociodemographic variables associated with access to alloSCT in Canada is very limited, several studies in the United States looking at factors impacting access to transplant have highlighted certain variables such as income, race, and location of residence as predictors in receiving transplants [3,4,5,6,7,8]. In contrast to the previous studies conducted in the United States with similar methodology, the findings from this study show slightly different results in terms of variables impacting access to transplant. In non-Quebec Canada, low-income level was not associated with inferior access to alloSCT as was seen in studies done in the United States. Given the difference in the funding of the health care system in Canada compared to the United States, this finding may suggest that Canada’s publicly funded universal health care insurance program is protective against socioeconomic barriers that would typically impact access to healthcare services.

In this study, we found that there were significant differences in the rates of alloSCT by province of residence, with residents of Alberta having the highest transplant rates. Higher transplant rates were also seen in British Columbia and Manitoba; with lower transplant rates seen in Saskatchewan, Ontario, Nova Scotia, and New Brunswick. The reasons for the regional variation observed amongst the provinces are unclear, but may include differences in practice patterns, availability of resources within each center, and provincial budgets set aside for health care. In one Canadian study examining geographical disparities in access to liver transplants, the evidence suggested that interprovincial disparities did exist and appeared to be related to factors that precede the transplant assessment process and not simply the distance of residence from the transplant center [2]. Rurality was not shown to be associated with inferior access to transplant in this study as had been previously shown in a Canadian study using population-based data to measure access to hematopoietic stem cell transplant in Manitoba [9].

The association between time period and probability of transplant observed in all three cohorts shows an increasing number of transplant procedures being performed as the years progress. This increase is possibly due to an increasing number of resources and infrastructure to support the demand and need for alloSCT. Other possibilities previously highlighted in an article by Majhail et al. [13] include an increased demand for alloSCT, an increased utilization of reduced-intensity regimens, as well as an increased availability and diversity of donors, thereby increasing access to alloSCT in patients in need of them.

**Figure 5:**
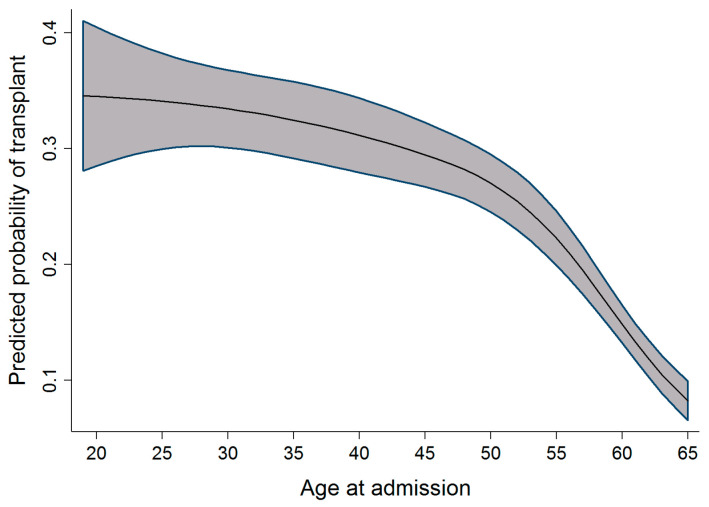
Relationship between age at admission and the probability of transplant (adult cohort).

**Figure 6:**
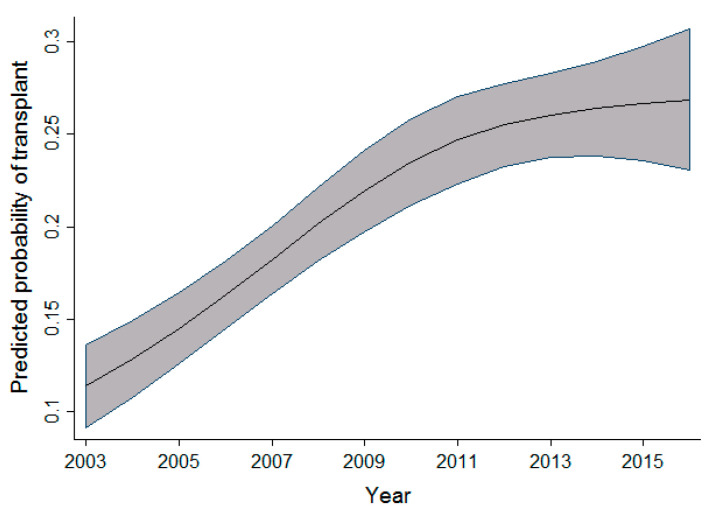
Relationship between time period and the probability of transplant (adult cohort).

As highlighted in Figure 3, Figure 4 and Figure 5, the relationship observed between age and the probability of receiving an alloSCT was an interesting finding in both the adult and pediatric cohorts. Overall, as shown in Figure 6, an increasing number of adults are receiving transplants as the years progress; however, the relative proportion decreases as people grow older. This decrease in the probability of receiving a stem cell transplant observed in the adult cohort past the age of 50 is mirrored in several medical interventions due to the presence of comorbidities that increase with age amongst several other factors, and a greater risk of complications associated with the myeloablative conditioning regimens associated with the receipt of an alloSCT.

In the pediatric cohort, the probability of receiving a transplant increases as the age of admission increases, with a peak at age 10 followed by a slight decline. A similar finding was observed in the study done on access to allogeneic transplants in pediatric patients with ALL by Truong et al. [10], in which a decline in transplant rates is seen in children over 10 years of age. One population-based study examining prognostic factors of childhood and adolescent AML survival found that patients diagnosed between 10 and 19 years of age were at a higher risk of death compared to those diagnosed before age 10 [14].

This increased risk of mortality observed within this age group has been reported in the literature, and studies in recent years have shown that adolescents and young adults diagnosed with malignancies often experience inferior health outcomes compared with younger and older cohorts for many reasons. In a review carried out by Trama et al. [15], a delay in the recognition and reporting of symptoms, reduced compliance with therapeutic regimens, and low enrollment in clinical trials were identified as some of the reasons for the trends observed within this age group.

Compared to the general population, the difference in transplant rate observed among visible minorities was not statistically significant. This finding is different from trends seen in some studies conducted in the United States, where non-Caucasians were observed to have lower rates of stem cell transplants compared to Caucasians [3,6]. Similarly, in comparing trends observed in stem cell transplantation with those seen in solid organ transplants, one retrospective cohort study conducted in Canada showed that access to kidney transplantation was significantly reduced for all ethnic groups compared to white Canadians [1]. When compared to non-indigenous patients, the difference in transplant rates observed among indigenous patients was not found to be statistically significant. This was an important finding given that a recent review carried out by Horrill et al. [16] showed indigenous populations faced significant barriers in accessing cancer care at an individual, systemic, and structural level.

There are several strengths and weaknesses to our study. This is the first study in Canada to examine some of the variables that impact access to allogeneic stem cell transplants in adult patients with AML at the national level. While some of our findings are consistent with those seen in previous studies examining access to stem cell transplants, other findings show trends different to those previously reported in the literature, some of which can be accounted for by differences in the funding of the healthcare system. 

Although our study was limited to individuals under the age of 65 diagnosed with AML, an increasing number of individuals over the age of 65 diagnosed with AML are recipients of alloSCTs due to the availability of reduced-intensity conditioning regimens that are better tolerated in the older adult population. As a result, further research is needed to include patients in this age group to understand factors that impact their access to receiving transplants. Furthermore, additional research is needed to understand the reasons behind the regional variation in rates of transplants observed among the provinces in Canada.

There are currently no Canadian guidelines outlining which patients with AML should receive an alloSCT, but there are several international guidelines publications that are widely used in Canada. We believe there is generally good consensus as to which patients with AML benefit from transplant, and that the finding that there is differential access to transplant is due to reasons other than interpretations of the medical literature.

The availability of a donor is a prerequisite for alloSCT. It is possible that donor availability might be different in different demographic groups. However, in an era of large unrelated donor registries and increased use of alternate donors (such as haploidentical donors), most patients will have an available donor. Thus, the impact of differential donor availability will be small. 

In summary, we found that in patients diagnosed with AML, rates of transplants varied depending on province of residence, with Alberta having the highest rates in the country. We also found an association between the time period and probability of transplants in all three cohorts observed. Transplant rates also appeared to vary depending on the age group of the individuals. However, differences in transplant rates among visible minorities, indigenous populations, and low-income groups were not found to be statistically significant.

## Figures and Tables

**Table 1:** Descriptive characteristics for the variables of interest.

Variables	Transplant Status	Statistics	Full Cohort	Pediatric Cohort	Adult Cohort
Age at admission	Transplant	n	1754	202	1552
		Median	47	9.5	49
		IQR	22	11	18
	No transplant	n	4365	359	4006
		Median	54	8	55
		IQR	21	12.08	15
		*p*-value	<0.01	0.03	<0.01
Proportion of visible minorities	Transplant	n	1745	199	1546
		Median	0.1	0.06	0.1
		IQR	0.23	0.26	0.22
	No transplant	n	4348	358	3990
		Median	0.09	0.1	0.09
		IQR	0.26	0.26	0.226
		*p*-value	0.59	0.27	0.33
Proportion of low-income families (after tax)	Transplant	n	1745	199	1546
		Median	6.5	6.2	6.5
		IQR	6	5.3	6
	No transplant	n	4348	358	3990
		Median	6.9	6.5	6.9
		IQR	6	6.2	6
		*p*-value	0.05	0.4	0.09
Proportion identifying as Indigenous	Transplant	n	1745	199	1546
		Median	0.02	0.02	0.02
		IQR	0.04	0.05	0.04
	No transplant	n	4348	358	3990
		Median	0.02	0.02	0.02
		IQR	0.04	0.04	0.04
		*p*-value	0.01	1	0.01

**Table 2:** Univariable and multivariable logistic regression models (Full cohort).

Variables of Interest	Categories	n	Univariable Models				Multivariable Model			
			Odds Ratio	95% Confidence Interval	*p*-Value	Overall *p*-Value	Odds Ratio	95% Confidence Interval	*p*-Value	Overall *p*-Value
Period	Continuous	6119	1.07	1.05–1.09	<0.01	<0.01	-	-	-	-
	RC spline1	-	-	-	-	-	1.16	1.11–1.21	<0.01	-
	RC spline2	-	-	-	-	-	0.91	0.86–0.96	<0.01	<0.01
Province	Alberta	861	Reference	-	-	-	-	-	-	-
	British Columbia	1005	0.66	0.54–0.80	0	-	0.71	0.58–0.86	<0.01	-
	Manitoba	293	0.70	0.53–0.91	0.01	-	0.71	0.53–0.94	0.02	-
	Newfoundland and Labrador	159	0.42	0.28–0.62	0	-	0.48	0.32–0.72	<0.01	-
	Nova Scotia	238	0.41	0.29–0.57	0	-	0.43	0.31–0.61	<0.01	-
	Nunavut	8	0.83	0.20–3.50	0.80	-	0.76	0.18–3.25	0.71	-
	Northwest Territories	7	0.55	0.11–2.87	0.48	-	0.95	0.17–5.33	0.95	-
	Ontario	3247	0.45	0.38–0.52	0	-	0.47	0.40–0.55	<0.01	-
	Prince Edward Island	29	0.85	0.39–1.81	0.67	-	0.85	0.39–1.88	0.70	-
	Saskatchewan	262	0.48	0.35–0.65	0	-	0.51	0.37–0.70	<0.01	-
	Yukon	10	2.08	0.58–7.42	0.26	<0.01	2.40	0.64–8.83	0.19	<0.01
Rurality	Urban	4906	Reference	-	-	-	-	-	-	-
	Rural	1192	0.94	0.82–1.09	0.42	0.42	-	-	-	-
Gender	Female	2859	Reference	-	-	-	-	-	-	-
	Male	3260	0.84	0.75–0.94	<0.01	<0.01	0.89	0.79–1.00	0.05	0.05
Age	Continuous	6119	0.98	0.98–0.98	0	<0.01	-	-	-	-
	RC spline1	-	-	-	-	-	1.02	1.01–1.03	0.01	-
	RC spline2	-	-	-	-	-	0.97	0.96–0.99	<0.01	-
	RC spline3	-	-	-	-	-	0.69	0.51–0.93	0.01	<0.01
Proportion of visible minorities	Continuous	6093	0.87	0.67–1.14	0.32	0.32	-	-	-	-
Proportion of low-income families (after tax)	Continuous	6093	0.99	0.98–1.00	0.02	0.01	-	-	-	-
Proportion identifying as Indigenous	Continuous	6093	0.97	0.49–1.90	0.92	0.92	-	-	-	-

**Table 3:** Univariable and multivariable logistic regression models (Pediatric cohort).

Variables of interest	Categories	n	Univariable Models				Multivariable Model			
			Odds Ratio	95% Confidence Interval	*p*-Value	Overall *P*-Value	Odds Ratio	95% Confidence Interval	*p*-Value	Overall *p*-Value
Period	Continuous	561	1.07	1.02–1.12	<0.01	<0.01	-	-	-	-
	RC spline1	-	-	-	-	-	1.20	1.05–1.37	<0.01	-
	RC spline2	-	-	-	-	-	0.86	0.72–1.02	0.08	<0.01
Province	Alberta	100	Reference	-	-	-	-	-	-	-
	British Columbia	94	1.06	0.60–1.89	0.84	-	-	-	-	-
	Manitoba	29	1.10	0.48–2.56	0.82	-	-	-	-	-
	Newfoundland and Labrador	14	0.63	0.18–2.13	0.4	-	-	-	-	-
	Nova Scotia	19	0.91	0.33–2.52	0.86	-	-	-	-	-
	Nunavut	4	1.56	0.21–11.57	0.66	-	-	-	-	-
	Ontario	265	0.78	0.48–1.25	0.30	-	-	-	-	-
	Prince Edward Island	7	2.09	0.44–9.83	0.35	-	-	-	-	-
	Saskatchewan	29	0.60	0.24–1.48	0.26	0.72	-	-	-	-
Rurality	Urban	445	Reference	-	-	-	-	-	-	-
	Rural	112	1.39	0.91–2.13	0.12	0.13	-	-	-	-
Gender	Female	258	Reference	-	-	-	-	-	-	-
	Male	303	0.88	0.62–1.24	0.47	0.47	-	-	-	-
Age	Continuous	561	1.03	1.00–1.06	0.05	0.05	-	-	-	-
	RC spline1	-	-	-	-	-	1.18	1.08–1.29	<0.01	-
	RC spline2	-	-	-	-	-	0.83	0.74–0.93	<0.01	<0.01
Proportion of visible minorities	Continuous	557	0.80	0.36–1.79	0.59	0.58	-	-	-	-
Proportion of low-income families (after tax)	Continuous	557	0.98	0.95–1.01	0.21	0.20	-	-	-	-
Proportion identifying as Indigenous	Continuous	557	2.60	0.63–10.77	0.19	0.19	-	-	-	-

**Table 4:** Univariable and multivariable logistic regression models (Adult cohort).

Variables of Interest	Categories	n	Univariable Models				Multivariable Model			
			Odds Ratio	95% Confidence Interval	*p*-Value	Overall *p*-Value	Odds Ratio	95% Confidence Interval	*p*-Value	Overall *p*-Value
Period	Continuous	5558	1.07	1.05–1.09	<0.01	<0.01	-	-	-	-
	RC spline1	-	-	-	-	-	1.15	1.10–1.20	<0.01	-
	RC spline2	-	-	-	-	-	0.92	0.87–0.97	<0.01	<0.01
Province	Alberta	761	Reference	-	-	-	Reference	-	-	-
	British Columbia	911	0.62	0.51–0.76	<0.01	-	0.67	0.54–0.82	<0.01	-
	Manitoba	264	0.66	0.49–0.88	<0.01	-	0.67	0.49–0.91	0.01	-
	Newfoundland and Labrador	145	0.40	0.67–0.61	<0.01	-	0.45	0.29–0.69	<0.01	-
	Nova Scotia	219	0.37	0.26–0.53	<0.01	-	0.39	0.27–0.56	<0.01	-
	Nunavut	4	0.45	0.05–4.39	0.50	-	0.37	0.04–3.68	0.40	-
	Northwest Territories	7	0.54	0.11–2.83	0.47	-	0.88	0.15–5.01	0.89	-
	Ontario	2982	0.42	0.36–0.50	<0.01	-	0.44	0.67–0.52	<0.01	-
	Prince Edward Island	22	0.64	0.26–1.58	0.33	-	0.64	0.25–1.63	0.35	-
	Saskatchewan	233	0.46	0.33–0.64	<0.01	-	0.49	0.35–0.69	<0.01	-
	Yukon	10	2.05	0.57–7.31	0.27	<0.01	2.31	0.62–8.59	0.21	<0.01
Rurality	Urban	4461	Reference	-	-	-	Reference	-	-	-
	Rural	1080	0.90	0.77–1.04	0.16	0.16	-	-	-	-
	Female	2601	Reference	-	-	-	Reference	-	-	-
	Male	2957	0.84	0.74–0.94	<0.01	0.03	0.89	0.79–1.00	0.06	0.06
Age	Continuous	5558	0.97	0.96–0.97	<0.01	<0.01	-	-	-	-
	RC spline1	-	-	-	-	-	1.00	0.97–1.03	0.87	-
	RC spline2	-	-	-	-	-	0.99	0.96–1.02	0.39	-
	RC spline 3	-	-	-	-	-	0.57	0.39–0.86	<0.01	<0.01
Proportion of visible minorities	Continuous	5536	0.88	0.67–1.17	0.38	0.38	-	-	-	-
Proportion of low-income families (after tax)	Continuous	5536	0.99	0.98–1.00	0.04	0.04	-	-	-	-
Proportion identifying as Indigenous	Continuous	5536	0.67	0.31–1.47	0.32	0.31	-	-	-	-

## Data Availability

The data presented in this study is available in this article.
